# Transient Overexpression of pVHL Mediated by Adenoviral Vector Injection in Pancreatic Tissue Decreases Blood Glucose Levels in a Hypercaloric Diet-Induced Mouse Model of Type 2 Diabetes Mellitus

**DOI:** 10.3390/ijms27104640

**Published:** 2026-05-21

**Authors:** Alma N. Díaz-Herreros, Elba Reyes-Maldonado, Erika Rosales-Cruz, Fernando Gómez-Chávez, Amaranta Sarai Valdez-Guerrero, Octavio Rodríguez-Cortés, Juan C. Cancino-Díaz, Mario E. Cancino-Díaz

**Affiliations:** 1 Laboratorio de Inmunología Aplicada, Departamento de Inmunología, Escuela Nacional de Ciencias Biológicas, Instituto Politécnico Nacional, Mexico City 11340, Mexico; nellyradio.s@gmail.com; 2 Centro de Investigación Científica Serendipia A.C., Tláhuac, Mexico City 13210, Mexico; 3 Laboratorio de Hematología, Departamento de Morfología, Escuela Nacional de Ciencias Biológicas, Instituto Politécnico Nacional, Mexico City 11340, Mexico; elreyes@ipn.mx (E.R.-M.); erosalesc@ipn.mx (E.R.-C.); 4 Laboratorio de Enfermedades Osteoarticulares e Inmunológicas, Sección de Estudios de Posgrado e Investigación, Escuela Nacional de Medicina y Homeopatía, Instituto Politécnico Nacional, Mexico City 07320, Mexico; fgomezch@ipn.mx; 5 Laboratorio de Bioquímica Aplicada, Escuela Superior de Medicina, Instituto Politécnico Nacional, Mexico City 11340, Mexico; amvaldezg@ipn.mx; 6 Laboratorio de Inflamación y Obesidad, Escuela Superior de Medicina, Instituto Politécnico Nacional, Mexico City 11340, Mexico; orodriguezc@ipn.mx; 7 Laboratorio de Inmunomicrobiología, Departamento de Microbiología, Escuela Nacional de Ciencias Biológicas, Instituto Politécnico Nacional, Mexico City 11340, Mexico; jcancinod@ipn.mx

**Keywords:** pVHL, HIF-1α, type 2 diabetes mellitus, pancreatic tissue, adenoviral vector, GLUT-1, GLUT-2, insulin, VHL–HIF-1α axis

## Abstract

The VHL–HIF-1α–VEGF axis regulates angiogenesis and metabolism. Beyond oncology, pVHL is essential for pancreatic β-cell function and is reduced in hypercaloric diet (HCD)-induced type 2 diabetes mellitus (T2DM). This study aimed to overexpress pVHL in pancreatic tissue and evaluate its effects on blood glucose levels and the expression of proteins related to glucose metabolism in the pancreas. HCD-induced diabetic C57BL/6 and BALB/c mice received a single intrapancreatic injection of an adenoviral vector (1 × 10^12^ viral particles) encoding the murine *Vhlh* gene (AdVHL) to induce transient pVHL overexpression. The glycemic delta (post-load glucose minus fasting) and net incremental area under the curve (niAUC) were determined on days 3, 6, 9, 12, and 15 post-treatment, as the peak in GFP overexpression (used as a surrogate reporter of transduction efficiency) was detected between days 9 and 12. Immunohistochemistry (IHC) and immunofluorescence (IF) were used to assess the expression of pVHL, HIF-1α, GLUT-1, GLUT-2, and insulin in pancreatic tissue. AdVHL treatment significantly decreased the glycemic delta and niAUC in mice with T2DM (*p* < 0.01). On day 15 after treatment, HIF-1α and GLUT-1 expression were markedly reduced in AdVHL-treated mice (*p* < 0.01), while GLUT-2 and insulin were significantly increased (*p* < 0.01). These results were reproduced in both mouse strains. Transient overexpression of pVHL in pancreatic tissue of mice with T2DM was associated with decreased glucose levels and changes in the expression of proteins related to glucose metabolism in the pancreas, resembling a healthier phenotype than that of mice with T2DM. These findings support an important functional role of the pVHL–HIF-1α axis in pancreatic physiology, provide a proof-of-concept for further mechanistic and translational studies, and implicate pVHL in the altered glucose metabolism observed in T2DM.

## 1. Introduction

Type 2 diabetes mellitus (T2DM) is a chronic, degenerative disease linked to multiple comorbidities. Its main features are high blood glucose levels, insulin resistance, and glucose intolerance. The global prevalence of diabetes is projected to increase from 537 million adults in 2025 to 783 million by 2045, with type 2 diabetes accounting for over 90% of all cases [1]. The causes of disrupted glucose metabolism and insulin resistance are complex and not fully understood; however, changes in pancreatic β-cell function have consistently been associated with disease progression. Accordingly, several studies have explored pancreatic development, with a particular focus on β-cells, since these are the only cells that produce insulin in adults [2]. Active areas of investigation include the physiology of β-cell precursors and the functional maturity and diversity of β-cells. Regeneration of pancreatic tissue—through β-cell proliferation, dedifferentiation, and transdifferentiation—is another area under active study [3].

The pancreas contains the islets of Langerhans, where β-cells produce insulin and coexist with α-, δ-, and ε-cells and pancreatic polypeptide (PP) cells, which release glucagon, somatostatin, ghrelin, and PP, respectively [1,2,4]. The islet is a highly vascularized endocrine tissue, and its blood supply is primarily regulated by the VHL–HIF-1α axis via VEGF, although this axis also regulates other metabolic pathways, including glycolysis. Of note, Von Hippel–Lindau disease was first described by Eugen von Hippel in 1895 and by Arvid Lindau in 1926, well before the molecular activity of the VHL–HIF-1α axis began to be characterized at the start of the 21st century. A germline mutation in the *VHL* gene causes the disease, which is characterized by benign and malignant tumors in organs such as the central nervous system, kidney, and pancreas [5,6].

Inactivation of the *Vhlh* gene in mice has demonstrated that pVHL plays important roles in the survival, proliferation, and differentiation of many cell types. *Vhlh* inactivation in the kidney results in the development of hemangioblastomas; in the liver, it leads to steatosis with alterations in glycolytic metabolism [7]. In humans with VHL mutations, cysts and tumors, including neuroendocrine tumors in the pancreas, have been detected [7,8,9]. In 2008, Zehetner et al. [10] generated *Vhlh*-deficient β-cells by crossing loxP-flanked *Vhlh* transgenic mice with Rip2-Cre mice. They found that inactivation of *Vhlh* enhanced lactate secretion and ATP production under low-glucose conditions but impaired glucose tolerance and insulin secretion under high-glucose stimulation [10]. A pivotal study, published in 2009, generated Pdx-1-CreER; VhlhLoxP/LoxP mice to specifically inactivate pVHL in adult β-cells. These mice developed a glucose-intolerant phenotype 6–8 weeks after tamoxifen treatment, which was accompanied by decreased insulin production and secretion, reduced GLUT-2 expression, and increased GLUT-1 expression and islet blood vessel density [11]. Together, these results indicate that β-cells from *Vhlh*-deficient mice fail to secrete insulin in response to glucose challenge. Similar findings were described by Cantley et al. in 2009 [12] and Choi et al. in 2011 [13], who also found a significant reduction in GLUT-2 and that high GLUT-1 expression is regulated by HIF-1α.

In summary, while several studies have demonstrated that genetic ablation of pVHL in pancreatic β-cells causes a marked alteration in glucose tolerance in mice, none have examined whether this protein is inactive in naturally diabetic mice. In a recent study, we examined pVHL expression in pancreatic islets from mice with a hypercaloric diet (HCD)-induced T2DM and observed a significant reduction in pVHL levels in HCD-T2DM mice compared to healthy controls [14]. In the present study, we demonstrated that transient overexpression of pVHL in pancreatic tissue of diabetic mice—via intrapancreatic injection of an adenoviral vector encoding the murine *Vhlh* gene (AdVHL)—led to a decrease in blood glucose levels, demonstrating that pVHL has an important functional role in the pancreas in maintaining glucose metabolic homeostasis.

## 2. Results

### 2.1. Efficient Transduction of AdGFP into Pancreatic Tissue

**Figure 1:**
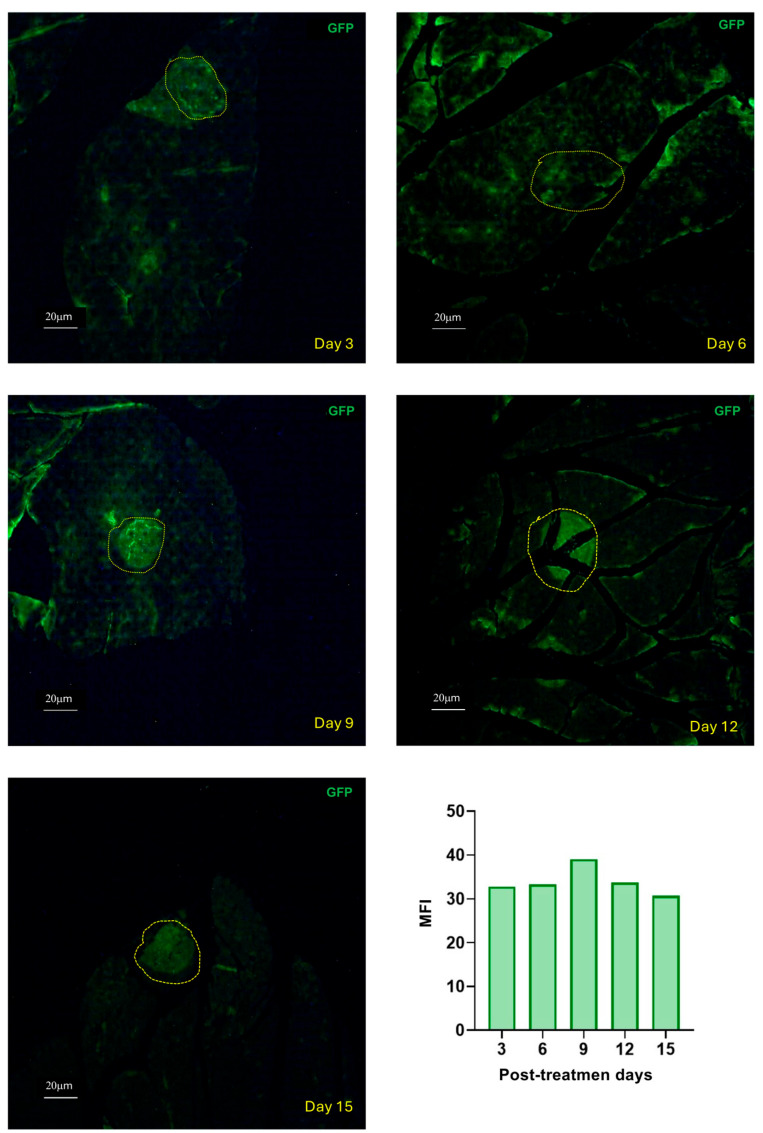
GFP production kinetics in pancreatic tissues. Healthy mice were treated via intrapancreatic injection with 1 × 10^12^ viral particles of AdGFP, and confocal analysis was performed on pancreatic tissues on days 3, 6, 9, 12, and 15 post-treatment to assess GFP production. Possible pancreatic islets are delineated with yellow circles. Scale bar = 20 µm. n = 1 mouse per time point.

To confirm that a recombinant adenovirus can transduce pancreatic tissue cells and direct the expression of a recombinant protein, we performed a single experiment injecting 1 × 10^12^ viral particles of AdGFP intrapancreatically into five C57BL/6 mice. GFP expression in pancreatic tissues was observed and analyzed using confocal microscopy on days 3, 6, 9, 12, and 15 post-inoculation. GFP was detected in pancreatic tissue, with peak expression observed in pancreatic islet cells on days 9 and 12 (Figure 1); however, a marker of beta cells is necessary to confirm this. Based on these results, we designed the experimental scheme shown in Table 1.

### 2.2. Transient Overexpression of pVHL in Pancreatic Tissues of Mice with T2DM Decreases Blood Glucose Levels

**Figure 2:**
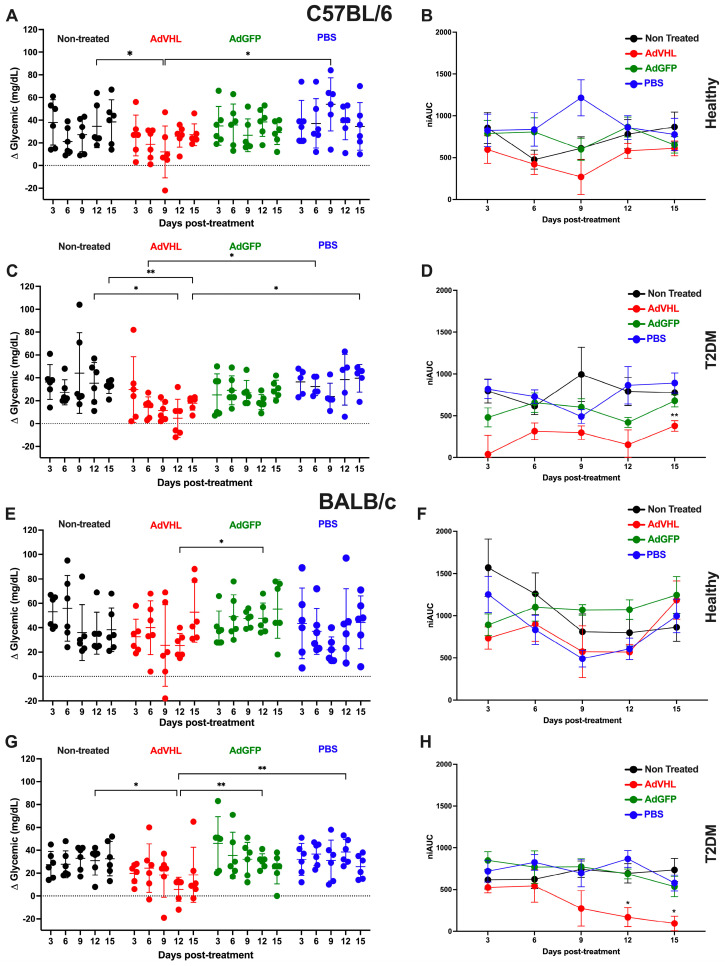
Treatment with AdVHL decreases blood glucose levels in C57BL/6 and BALB/c mice with T2DM. Healthy C57BL/6 mice (**A**,**B**) and those with T2DM (**C**,**D**), as well as healthy BALB/c mice (**E**,**F**) and those with T2DM (**G**,**H**), were treated via intrapancreatic injection with a single dose of AdVHL, AdGFP (transduction control), or PBS (treatment control). The glycemic delta (post-load blood glucose minus fasting blood glucose, measured 45 min after intragastric administration of 2 g/kg glucose) was measured on days 3, 6, 9, 12, and 15 post-treatment (**A**,**C**,**E**,**G**); niAUCs are shown in (**B**,**D**,**F**,**H**). All results are shown as the mean ± standard error of the mean. * *p* < 0.05; ** *p* < 0.01. For niAUC, * *p* < 0.05 and ** *p* < 0.01 show differences vs. the non-treated group. n = 6 independent mice per group, measured longitudinally at the five time points. Comparisons were made using two-way repeated-measures ANOVA followed by Tukey’s post hoc test.

It was previously demonstrated that pVHL production decreases in the pancreatic islets of HCD-induced T2DM mice [14]. In the present work, we investigated the effect of overexpressing this protein via intrapancreatic injection of AdVHL in T2DM and healthy mice (Figure 2). Figure 2C shows that C57BL/6 mice with T2DM treated with AdVHL (red circles) exhibited significantly (*p* < 0.01) lower glycemic delta values (15.6 ± 4.1 mg/dL) than untreated diabetic mice (black circles, 35.1 ± 2.7 mg/dL) and mice treated with AdGFP (green circles, 26.0 ± 2.0 mg/dL) or PBS (blue circles, 34.0 ± 2.8 mg/dL). Each reported value represents the mean ± SEM of 30 measurements (6 mice × 5 time points) per group. The greatest reduction in glycemic delta was observed on days 9 and 12 (*p* < 0.01), coinciding with the period of peak GFP expression (Figure 1). On day 12, 50% of the AdVHL-treated diabetic mice even showed negative glycemic delta values, indicating post-load blood glucose levels below fasting levels. Similarly, on day 12 the niAUC (Figure 2D) of mice with T2DM treated with AdVHL (236.7 ± 61 mg/dL × min) was lower than that of untreated diabetic mice (794.3 ± 60 mg/dL × min), AdGFP-treated diabetic mice (567.8 ± 50.4 mg/dL × min), and PBS-treated diabetic mice (758.7 ± 72.4 mg/dL × min) (*p* < 0.01).

Similar results were obtained with diabetic BALB/c mice treated with AdVHL (Figure 2G). Across the 15-day follow-up, these AdVHL-treated diabetic mice exhibited significantly (*p* < 0.01) lower glycemic delta values (17.3 ± 3.8 mg/dL) than untreated diabetic mice (30.3 ± 1.2 mg/dL) and mice treated with AdGFP (33.6 ± 3.6 mg/dL) or PBS (32.7 ± 2.2 mg/dL). This difference was even more pronounced when niAUCs were calculated (Figure 2H): the average niAUC across the 15 days for AdVHL-treated diabetic mice was 321 ± 91.7, whereas those for untreated, AdGFP-treated, and PBS-treated diabetic mice were 681.8 ± 27, 722.3 ± 53, and 737.3 ± 50.8, respectively. Although BALB/c mice are frequently described as having low susceptibility to HCD-induced T2DM, we and others have demonstrated that they do develop T2DM when fed an HCD formulated specifically for this strain, and the effect of pVHL observed in diabetic C57BL/6 mice was also observed in diabetic BALB/c mice.

The effect of pVHL on glucose metabolism was also studied in healthy non-diabetic mice of both strains. A significant alteration of glucose metabolism upon transient pVHL overexpression was also observed in non-diabetic C57BL/6 mice. The mean glycemic delta across the 15 days (Figure 2A) in AdVHL-treated healthy mice (22.0 ± 2.9 mg/dL) was lower than that of untreated mice (31.9 ± 3.3 mg/dL), AdGFP-treated mice (33.1 ± 2.7 mg/dL), and PBS-treated mice (40.4 ± 3.4 mg/dL) (*p* < 0.01). Analysis of the niAUCs yielded similar results. The mean niAUC of AdVHL-treated healthy C57BL/6 mice (495 ± 66 mg/dL × min) was lower than that of untreated healthy mice (717 ± 75 mg/dL × min), AdGFP-treated mice (742 ± 49 mg/dL × min), and PBS-treated mice (902 ± 79 mg/dL × min) (*p* < 0.001) (Figure 2B). Interestingly, in healthy mice, the glucose-lowering effect of pVHL overexpression was observed 3 days earlier (day 9) than in diabetic mice (day 12), likely because their healthy pancreas constitutively produces pVHL under baseline conditions. The effect of transient pVHL overexpression in the pancreas on lowering blood-glucose levels in healthy BALB/c mice was not significant; however, on day 9, four of the six mice showed the lowest glycemic deltas, and one of them was even negative (Figure 2E,F). The fact that the glucose-lowering effect of pVHL overexpression was observed in healthy C57BL/6 mice but not in healthy BALB/c mice is likely due to C57BL/6 mice being more susceptible to developing diabetes than BALB/c mice and having higher basal blood glucose levels.

### 2.3. AdVHL Treatment Reduces HIF-1α Expression in Pancreatic Tissues

**Figure 3:**
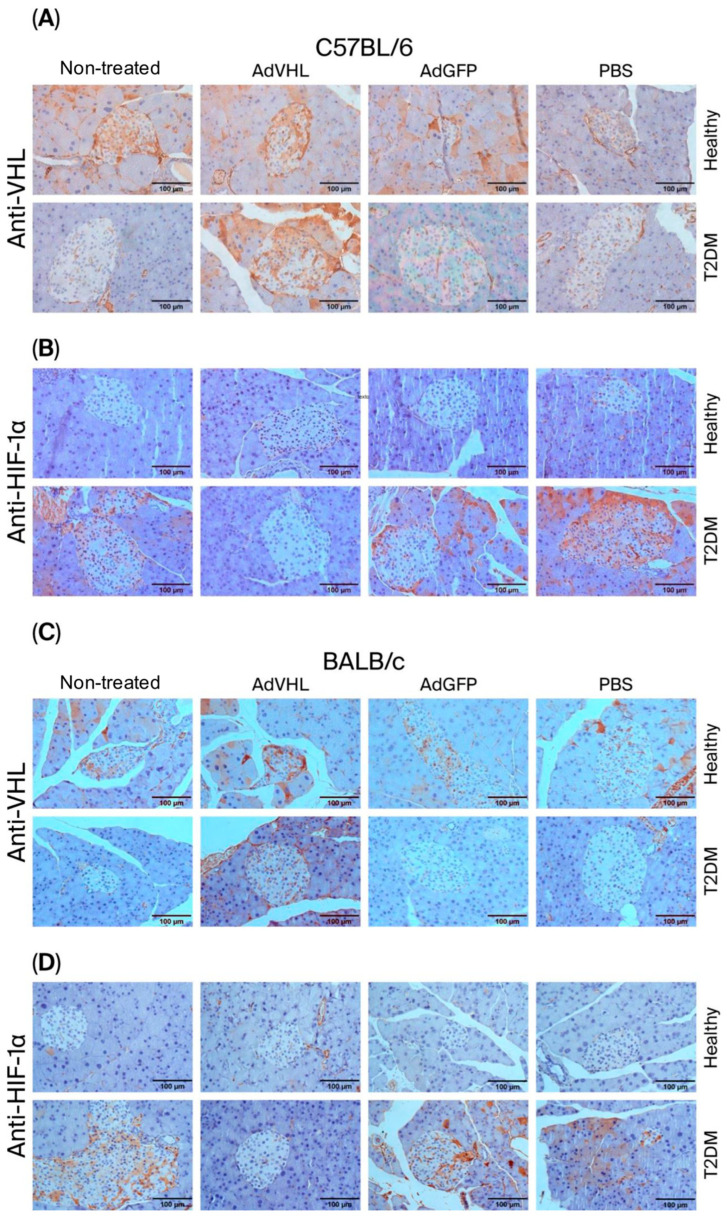
Transient overexpression of pVHL in pancreatic tissues of T2DM mice decreases HIF-1α expression. Immunohistochemistry was performed on day 15 post-treatment on pancreatic tissues from C57BL/6 (**A**,**B**) and BALB/c (**C**,**D**) mice with and without T2DM, treated with a single dose of AdVHL, AdGFP, or PBS, using anti-VHL (**A**,**C**) and anti-HIF-1α (**B**,**D**) antibodies. Detection was performed using biotinylated secondary antibodies and streptavidin–peroxidase, and incubation in diaminobenzidine, chromogenic (ochre color) and counterstained with hematoxylin and eosin. All images were captured at 40× magnification. Scale bar = 100 µm. Images are representative of n = 6 biological replicates per group, three non-overlapping 40× fields per section. Quantification of these images is shown in Figure 4.

**Figure 4:**
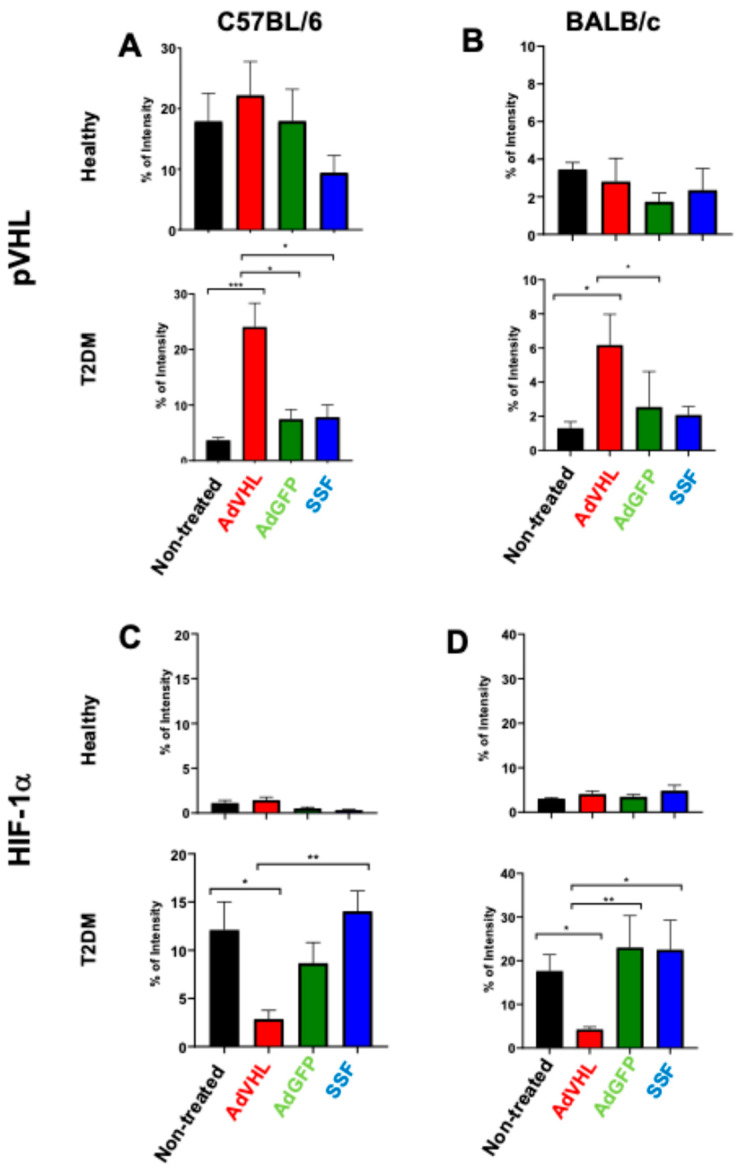
Quantitative analysis of VHL and HIF-1α expression in pancreatic tissues analyzed using immunohistochemistry. The mean percentage of positive-staining intensity (% of intensity) shown is from immunohistochemistry assays performed with anti-VHL (**A**,**B**) and anti-HIF-1α (**C**,**D**) antibodies on pancreatic tissues from healthy and T2DM C57BL/6 and BALB/c mice, untreated or treated with a single dose of AdVHL, AdGFP, or PBS, and analyzed 15 days post-treatment. The percentage of intensity was quantified using ImageJ software v1.54p. Data are shown as individual values, mean ± SEM; 6 biological replicates per group, 3 fields per section, n = 18. One-way ANOVA with Tukey’s post hoc test vs. the untreated group of the same strain and on the same diet. * *p* < 0.05; ** *p* < 0.01; *** *p* < 0.001.

It is well established that pVHL regulates HIF-1α activity through ubiquitination and proteasomal degradation. Qualitative analysis of Figure 3A shows that pVHL was detected at high levels in pancreatic tissues from healthy C57BL/6 mice but not in T2DM mice. The mean percentage of positive-staining intensity (% of intensity) for pVHL in healthy mice was 17.9 ± 4.63, whereas in diabetic mice it was 3.6 ± 0.49 (*p* < 0.01) (Figure 4A); conversely, HIF-1α was highly expressed in T2DM mice but not in healthy mice (Figure 3B). The % of intensity of HIF-1α in diabetic mice was 12.1 ± 2.91, and in healthy mice, it was 1.1 ± 0.30 (Figure 4C). These findings are consistent with our previously published data [14]. The novel finding of this work is that when the mice received a single dose of AdVHL (or AdGFP), at 15 days post-treatment, there was an increase in pVHL in both healthy and T2DM mice (Figure 3A), yielding a % of intensity of approximately 22.0 in both cases (Figure 4A), whereas HIF-1α was undetectable in both groups (Figure 3B), yielding a % of intensity below 3.0 in both groups (Figure 4C). In contrast, AdGFP- and PBS-treated mice displayed the same pattern as untreated controls (Figure 3B), and the % of intensity in these groups was around 10.0 (Figure 4C). Similar results were obtained in BALB/c mice: pVHL was reduced in T2DM mice (% of intensity = 1.29 ± 0.38) compared to healthy controls (% of intensity = 3.44 ± 0.37) (Figure 3C and Figure 4B), and treatment with AdVHL decreased HIF-1α expression (Figure 3D), yielding a % of intensity of 4.26 ± 0.58 (Figure 4D).

### 2.4. AdVHL Treatment Decreases GLUT-1 Expression in Pancreatic Tissues from Mice with T2DM

**Figure 5:**
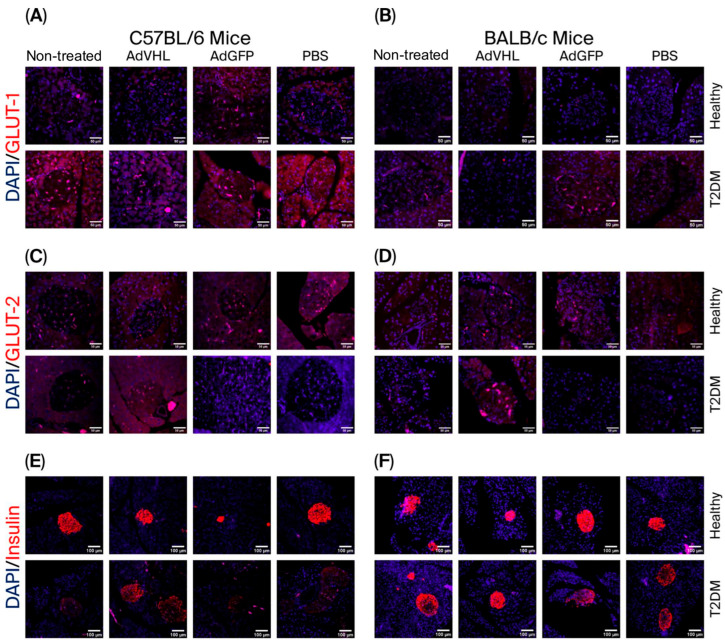
Overexpression of pVHL in pancreatic tissues of T2DM mice decreases GLUT-1 and induces GLUT-2 and insulin overexpression. Pancreatic tissues from C57BL/6 (**A**,**C**,**E**) and BALB/c (**B**,**D**,**F**) mice with and without T2DM, treated with a single dose of AdVHL, AdGFP, or PBS, were analyzed 15 days post-treatment using confocal microscopy; anti-GLUT-1 (**A**,**B**), anti-GLUT-2 (**C**,**D**), and anti-insulin (**E**,**F**) primary antibodies; and Alexa Fluor 594–anti-goat IgG secondary antibody (red). DAPI (Blue). All images were captured at 40× magnification. Scale bar = 100 µm. Images are representative of n = 6 biological replicates per group, three non-overlapping 40× fields per section. Quantification is shown in Figure 6.

**Figure 6:**
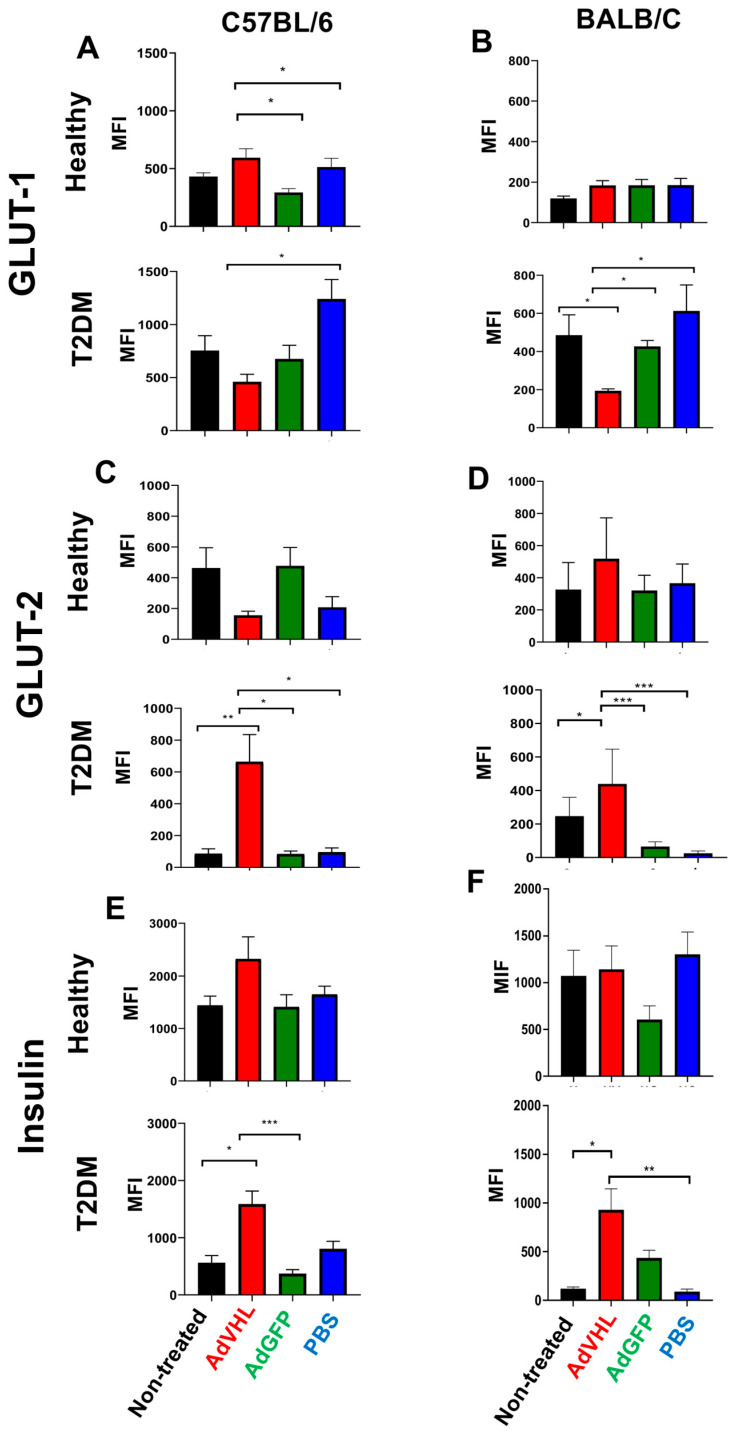
Quantitative analysis of GLUT-1, GLUT-2, and insulin expression in pancreatic tissues using immunofluorescence. Mean fluorescence intensity (MFI) from immunofluorescence assays performed with anti-GLUT-1 (**A**,**B**), anti-GLUT-2 (**C**,**D**), and anti-insulin (**E**,**F**) antibodies on pancreatic tissues from healthy and T2DM C57BL/6 and BALB/c mice, untreated or treated with a single dose of AdVHL, AdGFP, or PBS after 15 days. MFI was quantified using ImageJ software. Data are shown as mean ± SEM; 6 biological replicates per group, 3 islets per section, n = 18. One-way ANOVA with Tukey’s post hoc test vs. the untreated group of the same strain and under the same diet. * *p* < 0.05; ** *p* < 0.01; *** *p* < 0.001.

GLUT-1 is a glucose transporter whose expression in pancreatic β-cells is regulated by HIF-1α. Analysis of GLUT-1 expression yielded results consistent with those observed for HIF-1α. GLUT-1 expression was low in pancreatic tissues of healthy C57BL/6 (MFI = 430 ± 32.92) and BALB/c (MFI = 118 ± 12.12) (Figure 5A,B and Figure 6A,B) mice, but markedly elevated in T2DM mice of both strains (MFI = 752 ± 142 for C57BL/6 and MFI = 484.5 ± 107.3 for BALB/c). Quantitative analysis also showed that GLUT-1 mean fluorescence intensity (MFI) was approximately two-fold higher in T2DM than in healthy mice, a difference that was more pronounced in BALB/c than in C57BL/6 mice (*p* < 0.01) (Figure 6A,B). When mice were treated with a single dose of AdVHL or AdGFP and analyzed on day 15 post-treatment, we observed that AdVHL treatment significantly reduced GLUT-1 expression in T2DM mice of both strains. In C57BL/6 mice, the MFI was 752.7 ± 142 in untreated mice, 459.3 ± 71.61 in AdVHL-treated mice, 674.0 ± 130.1 in AdGFP-treated mice, and 1240 ± 184.2 in PBS-treated mice; in BALB/c mice, the MFI was 484.5 ± 107.3 in untreated animals and 192.9 ± 10.73 in AdVHL-treated animals (*p* < 0.001), whereas GLUT-1 remained high in AdGFP-treated (MFI = 425.5 ± 32.11) and PBS-treated mice (MFI = 611.4 ± 137.2) (Figure 5A,B and Figure 6A,B).

### 2.5. AdVHL Treatment Induces GLUT-2 and Insulin Overexpression in Pancreatic Tissues from Mice with T2DM

Because GLUT-2 and insulin are positively regulated by pVHL, we investigated their expression in pancreatic tissues from T2DM mice treated for 15 days after a single dose of AdVHL (or AdGFP). The expression for GLUT-2 was significantly higher in pancreatic islets of healthy C57BL/6 mice (MFI = 462.3 ± 132.7) than in those of T2DM mice (MFI = 85.42 ± 30.81). This reduction was significantly reversed by AdVHL treatment, with MFI rising from 85.42 ± 30.81 to 663.8 ± 170.9, whereas AdGFP and PBS had no effect (MFI remained at 83.77 ± 19.07 and 95.28 ± 26.93, respectively) (*p* < 0.001; Figure 5C and Figure 6C). In BALB/c mice, the results were similar: AdVHL treatment increased GLUT-2 expression in diabetic mice (MFI rising from 246.4 ± 35.58 to 438.7 ± 62.62), whereas the AdGFP and PBS treatments had no effect (MFI values remained below 70) (Figure 5D and Figure 6D). Regarding insulin expression, transient overexpression of pVHL in the pancreas increased insulin expression. The insulin MFI was lower in T2DM C57BL/6 mice (MFI = 559.6 ± 127.9) than in healthy mice (1440 ± 177.9), and was significantly higher in AdVHL-treated mice (1587 ± 228.3) than in untreated, AdGFP-treated (369.5 ± 72.92), or PBS-treated (803.8 ± 134) T2DM mice (*p* < 0.001; Figure 5E and Figure 6E). In BALB/c mice, this effect of AdVHL was also observed: the MFI in T2DM mice was 118.8 ± 17.69 and increased to 929.0 ± 216.4 after AdVHL treatment (*p* < 0.01; Figure 5F and Figure 6F). These results suggest that loss of pVHL activity in the pancreas is associated with decreased insulin production. 

## 3. Discussion

The molecular and cellular mechanisms that cause T2DM are not fully understood; however, it is well established that β-cells play a central role in T2DM as these cells progressively lose their ability to produce insulin during disease development. Pancreatic cells originate from ductal progenitors that differentiate in a coordinated manner into endocrine and exocrine tissues. β-cell progenitors are characterized by expression of transcription factors including Pdx1, Ptf1a, Ngn3, Nkx6.1, and Sox9 [15,16], which are required for differentiation into mature, insulin-producing β-cells. Mature β-cells express Pdx1, MafA, NeuroD1, Ins1/2, Nkx2.2, and Cdkn2a [17,18]. In pancreatic tissues from adults with T2DM, expression of transcription factors associated with undifferentiated β-cells has been observed, suggesting that β-cells may undergo dedifferentiation, transdifferentiation, or cellular senescence—states in which insulin production ceases [19,20].

Current pharmacological approaches to T2DM, including glucagon-like peptide-1 receptor (GLP-1R) agonists, glucose-dependent insulinotropic polypeptide receptor (GIPR) agonists, and dual agonists such as tirzepatide, act on downstream insulin-secretion pathways [21,22]. In contrast to these incretin-based therapies, in the present work, we showed that transient overexpression of pVHL in pancreatic tissue via AdVHL decreased blood glucose levels in HCD-induced T2DM mice and promoted insulin expression. This work was undertaken based on our prior finding of decreased pVHL activity in the pancreatic tissues of HCD-induced T2DM mice [14] and on the observation that deletion of the *Vhlh* gene in mature β-cells results in impaired glucose tolerance [10,11,12,13]. Our results show that transient overexpression of pVHL in the pancreatic tissues of diabetic mice was accompanied by a decrease in HIF-1α expression and a substantial increase in insulin production, suggesting that pVHL could facilitate increased insulin production in pancreatic tissues. However, this model represents early-stage diabetes and the observation window was limited to 15 days.

Our observations align with the findings reported by Soggia et al. (2014) [23]. They showed that HIF-1α expression is high during early pancreatic development (days 10.5–12.5 post-coitum) and disappears at maturity (day 17.5), while insulin-1 mRNA expression is low at day 12.5 and high at day 17.5 [23]. We observed that pVHL and HIF-1α showed inverse expression patterns: pVHL predominated in healthy pancreatic tissue, where insulin levels are high, whereas HIF-1α predominated in diabetic mice. Sox9 is a transcription factor that is highly expressed in β-cell precursors but not in mature β-cells. *Vhlh*-knockout mice generated using Sox9-Cre to delete *Vhlh* exclusively in precursor cells showed low insulin expression and reduced expression of mature β-cell genes, including Pdx1, Neurod, Nkx6.1, ZnT-8, and Pcsk1 [23]. These findings suggest that pVHL may be necessary for maintaining insulin production in pancreatic tissues.

Another functional link between pVHL and Sox9 was shown by Lynn et al. in 2013 [24]. When *Vhlh* was deleted in mature β-cells, high Sox9 levels were observed. Moreover, when Ins-1 and MIN6 cells and mouse and human islets were kept under hypoxic conditions (2% O_2_), Sox9 was overexpressed along with HIF-1α-regulated proteins (GLUT1, VEGFA, and PDK1), while the levels of mature β-cell proteins (PDX-1, NKX6.1, MAFA, and GLUT2) were decreased [24]. Taken together, these observations suggested that the transition from mature to immature β-cell states may occur under hypoxic conditions (e.g., ischemia) and may be biochemically driven by high glucose levels and the resulting inflammation. Based on our findings and those of other authors, we propose that the transition from a mature to an immature state in β-cells in diabetic subjects could be reversed by AdVHL treatment, as pVHL inactivates HIF-1α and may also lower Sox9 expression; however, additional experiments are required to confirm this. Notably, Puri et al. in 2024 also reported that Sox9 is expressed at low levels in mature β-cells, where it plays an important role in alternative splicing and controlling the insulin secretory pathway [25].

In most studies where *Vhlh* was deleted in β-cells, increased GLUT-1 and decreased GLUT-2 and insulin expression were reported [12]. Consistent with these findings, we observed that GLUT-2 was present in healthy pancreatic tissues but reduced in diabetic mice where pVHL expression was also low. Additionally, GLUT-2 expression increased in diabetic mice that transiently overexpressed pVHL. Similar results were reported by Choi et al. in 2011, who observed improved glucose tolerance and restoration of GLUT-2 in *Vhlh*-deleted β-cell mice treated with human recombinant erythropoietin (EPO) [13]. EPO is produced by kidney cells, hepatocytes, and brain and cardiac cells [26,27]; its receptor is present on β-cells [28]. EPO promotes pro-survival and proliferative signaling [29] and exerts cytoprotective effects [30]. Since both EPO and our transient pVHL treatment induced GLUT-2 expression and decreased blood glucose levels, we propose that high GLUT-2 expression may also enhance the glucose-sensing capacity of pancreatic tissue, thereby boosting insulin production. GLUT-2, unlike GLUT-1, has a low glucose affinity (high Km) but a high transport capacity, enabling β-cells to sense and transport glucose in proportion to blood glucose concentration.

Beyond the GLUT-2/glucose-sensing mechanism described above, hyperglycemia and inflammation are also known to induce endoplasmic reticulum (ER) stress in β-cells. ER stress activates the unfolded protein response (UPR), which involves ATF6, IRE1α, and PERK. β-cells can recover through an adaptive UPR; however, when ER stress becomes chronic in diabetes, the UPR turns maladaptive, leading to irreversible apoptosis [31]. Misfolded proteins can also be eliminated by autophagy, which is negatively regulated by the mechanistic target of rapamycin (mTOR) complex, with the regulatory-associated protein of mTOR (RAPTOR) playing a key role. Notably, in clear cell renal carcinoma, RAPTOR can be ubiquitinated by pVHL and broken down by the proteasome, thereby initiating autophagy [32]. The low pVHL levels observed in the pancreatic tissues of diabetic mice suggest that, without pVHL, RAPTOR degradation may be impaired, potentially blocking mTOR-mediated autophagy, which may drive apoptosis of pancreatic cells, a process strongly associated with diabetes. Based on the above, we propose that in our AdVHL-treated diabetic mice, RAPTOR degradation may increase, thereby activating autophagy and potentially alleviating pancreatic ER stress and supporting pancreatic function; we also propose that pVHL may regulate other proteins involved in the UPR pathway. However, all these hypotheses require direct experimental validation. We emphasize that the RAPTOR–mTORC1–autophagy link described above is based on extrapolation from renal cancer biology and was not directly tested: we did not measure RAPTOR or mTORC1 activity, UPR markers, or autophagy flux in pancreatic tissue. This mechanism should therefore be regarded as a speculative hypothesis to be tested in dedicated mechanistic studies, rather than as a conclusion of the present work.

Our results show that a transient overexpression of pancreatic pVHL in mice with T2DM was accompanied by increased insulin and GLUT-2 expression and reduced expression of GLUT-1, which was similar to the observations in the pancreatic tissue of non-diabetic mice.

## 4. Materials and Methods

### 4.1. Animal Model of Type 2 Diabetes Mellitus

This study was conducted in accordance with the guidelines of the Ethics Committee of the Escuela Nacional de Ciencias Biológicas (ENCB, IPN) (approval No. Z00-001-2021). Female C57BL/6 and BALB/c mice aged 8–10 weeks were acquired from the animal facility of UAM Xochimilco, Mexico City, and certified as specific-pathogen-free. Mice of both strains underwent a 6-week acclimatization period on standard chow (Labdiet 5001) until they reached 4 months of age.

The T2DM mouse model was induced using a hypercaloric diet as described by Díaz-Herreros et al. 2025 [14] and 2026 [33], and Appiakannan et al. 2020 [34]. Strain-specific hypercaloric diets (HCDs) were produced for each strain. A total of 96 mice were used: 48 C57BL/6 and 48 BALB/c. Twenty-four C57BL/6 mice were fed a standard diet (SD; Labdiet 5001) with ad libitum access and served as the healthy control group. The remaining 24 C57BL/6 mice were fed the strain-specific HCD for 10 weeks to induce T2DM. Similarly, 24 BALB/c mice were fed the strain-specific HCD for 10 weeks to induce T2DM, while the remaining 24 BALB/c mice were fed a standard diet (SD; Labdiet 5001) ad libitum and served as the healthy control group. For hydration, BALB/c mice received a 30% (*w*/*v*) sucrose solution, and C57BL/6 mice received a 20% (*w*/*v*) sucrose solution, both prepared from commercial sucrose (Sigma-Aldrich, St. Louis, MO, USA; Cat. S0389), dissolved in autoclaved drinking water, as an additional caloric source. Mice fed the SD (group of healthy mice) received sucrose-free autoclaved drinking water ad libitum. Fasting body weight was monitored weekly. Blood glucose levels were measured using an Accu-Chek Instant glucometer (Roche Diagnostics, Indianapolis, IN, USA) from tail vein blood samples collected after a 5 h fasting period beginning at 09:00 h.

The approximate calorie intake of each diet was as follows: the SD contained 3.35 Cal/g; the HCD for BALB/c contained 4.89 Cal/g, and the HCD for C57BL/6 contained 4.31 Cal/g. Each milliliter of water with 30% sucrose provided 1.2 calories, and 20% sucrose provided 0.8 calories. The diabetic C57BL/6 mice consumed an average of 71.45 ± 11.11 g of the HCD and 217.2 ± 20.9 mL of 20% sucrose for a total estimated caloric intake of 481.7 ± 47.8 kcal. The diabetic BALB/c mice consumed 41.89 ± 4.49 g of the HCD and 71.64 ± 11.23 mL of 30% sucrose for a total estimated caloric intake of 291.17 ± 35.42 kcal. The healthy C57BL/6 mice consumed an average of 108.3 ± 2.3 g of the SD and 131.2 ± 5.9 mL of water (362.8 ± 7.7 kcal in total), and the healthy BALB/c mice consumed 75.14 ± 2.3 g of the SD and 77.64 ± 3.9 mL of water (251.71 ± 7.7 kcal in total).

The mice were monitored for 10 weeks, with body weight and fasting glucose levels assessed weekly. Serum insulin, triglycerides, and cholesterol were also measured at the end of the 10-week period. The fasting glucose levels of C57BL/6 mice fed the HCD reached 142.9 ± 10.34 mg/dL, compared to 128.7 ± 3.3 mg/dL in C57BL/6 mice fed the SD. BALB/c mice fed the HCD also showed significant hyperglycemia (116.4 ± 5.4 mg/dL) compared to the BALB/c SD controls (85.14 ± 2.53 mg/dL). The body weight in C57BL/6 mice increased significantly from week 5 onward (27.2 ± 3.62 g on HCD vs. 22.8 ± 5.4 g on SD; *p* < 0.05). The C57BL/6 mice fed the HCD showed increased serum insulin (35.15 ± 15.94 pg/dL, *p* < 0.05) and triglyceride (410.6 ± 119.5 mg/dL, *p* < 0.01) levels compared to mice fed the SD (insulin: 11.16 ± 3.25 pg/dL; triglycerides: 171.10 ± 15.99 mg/dL). The HCD-fed BALB/c mice also showed increased serum insulin (47.12 ± 13.94 pg/dL, *p* < 0.05) and triglyceride (278.8 ± 95.5 mg/dL, *p* < 0.01) levels at the end of week 10 compared to mice fed the SD (insulin: 11.26 ± 7.85 pg/dL, triglycerides: 84.40 ± 12.39 mg/dL). Serum cholesterol levels also increased in HCD-fed mice (134.45 ± 24.11 mg/dL) compared to SD-fed mice (82.34 ± 21.68 mg/dL), but this was only seen in the C57BL/6 mice. The Homeostatic Model Assessment of Insulin Resistance (HOMA-IR) index was also determined; in HCD-fed mice, the HOMA-IR index was elevated (13.53 ± 1.8) compared to that of mice fed the SD (2.36 ± 1.15; *p* < 0.01). Furthermore, histological analysis revealed that HCD-fed mice at week 10 presented hepatic steatosis and adipocyte hyperplasia. Taken together, these findings indicate that both C57BL/6 and BALB/c mice fed the strain-specific HCD developed T2DM, whereas SD-fed mice remained metabolically healthy.

### 4.2. Expansion of Adenoviral Vectors

Two adenoviral vectors were used: AdVHL, which harbors the murine *Vhlh* gene and is capable of expressing recombinant murine pVHL, and AdGFP, which carries the green fluorescent protein (*GFP*) gene. Both vectors were generated as previously described [35]. Briefly, expansion was performed in HEK-293 cells using Dulbecco’s Modified Eagle Medium (DMEM) supplemented with 10% fetal bovine serum (FBS) and 1% antibiotics. Cells at 90–100% confluency were infected with AdVHL or AdGFP at a multiplicity of infection (MOI) of 0.1 and re-fed with DMEM containing 2% FBS for 7 days. The total contents of the culture flasks were collected, and the cells were lysed with 0.2% NP-40 buffer for 10 min, followed by 3 freeze–thaw cycles (−70 °C/37 °C). Cell debris was removed by centrifugation at 10,000 rpm for 10 min. The supernatant was mixed with a 1/2 volume of 20% polyethylene glycol (PEG) in 2.5 mM NaCl, incubated on ice for 1 h, and centrifuged to recover the viral pellet, which was then resuspended in 5 mL of CsCl (1.10 g/mL).

Adenoviruses were purified by CsCl density gradient ultracentrifugation (TH641 rotor, 20,000 rpm, 2 h, 20 °C) using a step gradient of 1.4 g/mL/1.30 g/mL/1.10 g/mL CsCl. The opalescent band between the 1.4 and 1.3 g/mL gradients was collected and passed through a Sepharose column; fractions were collected based on optical density at 260 nm. This procedure was repeated three times.

### 4.3. Treatment of Diabetic and Non-Diabetic (Healthy) Mice

After 10 weeks of dietary feeding and confirmation of T2DM as described in Díaz-Herreros et al. 2026 [33], the 24 mice with T2DM from each strain (C57BL/6 and BALB/c) were organized into 4 subgroups of 6 mice each. One subgroup (no treatment) served as the untreated control. A second subgroup (AdVHL) received a dose of 1 × 10^12^ viral particles of AdVHL via the intrapancreatic route. A third subgroup (AdGFP) received a dose of 1 × 10^12^ viral particles of AdGFP, and a fourth subgroup received 40 µL of phosphate-buffered saline (PBS) as the treatment-vehicle control. The glycemic delta and the net incremental area under the curve (niAUCs) were determined on days 3, 6, 9, 12, and 15 post-administration in all 6 mice of each subgroup. A parallel set of subgroups for each strain, fed a control diet (healthy mice), was also treated with AdVHL, AdGFP, or PBS, or left untreated (see Table 1) [33].

### 4.4. Surgical Intrapancreatic Administration of the Adenoviral Vector

The mice were fasted for 3 h before surgery. The midabdominal area was depilated. Each mouse was anesthetized with sodium pentobarbital (70 mg/kg intraperitoneally). Once sedation was confirmed (≈10 min post-injection), a 2 cm longitudinal incision was made from the xiphoid cartilage, separating the dermal and muscular planes. The pancreas was identified by anatomical landmarks, and the viral vector was injected directly into the pancreatic parenchyma at a dose of 1 × 10^12^ viral particles in 40 µL using a 0.3 mL insulin syringe (the PBS group received 40 µL of saline only). Dexamethasone (0.15 mg/kg) and 20 µL saline (intraperitoneal) were administered to prevent inflammation and fluid imbalance. The muscular plane was closed with 4–6 interrupted sutures using 4-0 polyglycolic acid sutures, and the dermal plane was closed with 4-0 nylon running sutures. The mice were placed under an incandescent lamp for post-anesthetic recovery (≈1 h).

### 4.5. Determination of Glycemic Delta and Net Incremental Area Under the Curve (niAUC)

The glucose tolerance test (GTT) involves measuring blood glucose at 0, 15, 30, 60, and 120 min after administration of a 2 g/kg body weight glucose bolus to the animal. Due to the large number of animals in this study (96 mice), to reduce the cost of test strips and the number of tail-vein punctures per animal, and given the limited number of technicians involved, we measured blood glucose at only two time points to study the effect of pVHL on glucose metabolism. We refer to this parameter as the glycemic delta, which is conceptually similar to the incremental peak or delta glucose peak described in recent studies of individual responses to carbohydrates [36]. Fasting baseline blood glucose (time 0) was determined, and blood glucose was measured again 45 min after an intragastric load of 2 g/kg body weight of glucose. We chose the 45 min post-load time point because, on average, in mice, glucose levels begin to decline at this time point due to the compensatory effect of insulin, and we considered these two points the most representative of a GTT. From these two values, we calculated the glycemic delta and the niAUC of the resulting two-point curve, as described below. The glycemic delta (mg/dL) was defined as the post-load glucose value minus the fasting glucose value for the same animal on the same day, with smaller (or negative) deltas indicating better short-term control of the post-load glucose excursion; it provides an internally normalized, within-animal index of glucose handling ability that is less sensitive to day-to-day variation in baseline glucose levels than absolute post-load values. Areas under the curve (niAUCs) for post-challenge glucose were calculated using the trapezoidal approximation of deviations from the baseline fasting glucose to glucose at 45 min post-challenge. The results are expressed as niAUC (mg/dL × min).

### 4.6. Immunohistochemistry

Immunohistochemical analysis was performed to measure VHL and HIF-1α expression in pancreatic tissues collected on day 15 post-treatment. The histological sections (3 µm) were mounted on electrocharged slides. Deparaffinization was performed by sequential immersion in xylene, xylene/absolute ethanol (1:1), absolute ethanol, 96° ethanol, 70° ethanol, and distilled water. Antigen retrieval was performed in a pressure cooker with 1X citrate buffer for 18 min. Non-specific blocking was performed using an ASE blocking solution (50 mM glycine, 0.05% Tween-20, 0.1% Triton-X-100, 1% BSA (Sigma-Aldrich, Cat. A7906), and 2% FBS in PBS (Gibco, Thermo Fisher Scientific, Waltham, MA, USA, Cat. 10010-023)) for 30 min at room temperature. After three 5 min PBS washes, the slides were incubated with the following primary antibodies at 37 °C for 1 h in a humidified chamber: anti-VHL (Novus Biologicals, Littleton, CO, USA; NB100-41384, 1:100) and anti-HIF-1α (Novus Biologicals, NB100-479, 1:100). Detection was performed using the Histostain kit (Invitrogen) with biotinylated secondary antibodies and streptavidin–peroxidase, followed by incubation in a DAB (diaminobenzidine) chromogenic solution (Biocare Medical, Concord, CA, USA) for 50–60 s. The sections were counterstained with hematoxylin and eosin, mounted with resin, and examined by conventional light microscopy.

For quantitative analysis, one histological section of pancreas per mouse was analyzed, and three fields were captured per section. Quantification was performed in Fiji/ImageJ v1.54p using the Color Deconvolution function (Channel 4—Brown) with a threshold of 11–250. The results are expressed as the percentage of positive-staining intensity in the islets of Langerhans. Quantification was performed on 6 mice per treatment group (one section per animal, and three non-overlapping 40× fields were captured per section, totaling 18 fields per group per time point, n = 18). The pancreas samples were collected on day 15 post-treatment. Image acquisition and quantitative analysis were not performed under blinded conditions; all comparisons were made within the strain, with the same diet, and at matched time points, and outliers were evaluated using Grubbs’ test (α = 0.05), which was applied to each biological replicate set before statistical comparison, with no more than one value per group excluded.

### 4.7. Immunofluorescence

Expression levels of insulin, GLUT-1, and GLUT-2 were determined by immunofluorescence using the same pancreatic tissues used to measure pVHL and HIF-1α expression (day 15 post-treatment). Deparaffinization and antigen retrieval were performed as described above (citrate buffer, pH 6.0; Invitrogen, Carlsbad, CA, USA; Cat. 005000). After blocking and primary antibody incubation (anti-GLUT-1, anti-GLUT-2, anti-insulin; 1 h, 37 °C), the slides were incubated with Alexa Fluor 594–anti-goat IgG secondary antibody (30 min, room temperature), followed by DAPI staining (20 min in the dark). The preparations were examined using confocal microscopy. Quantitative analysis was performed in Fiji/ImageJ using the Split Channels function to isolate antibody-positive signals, and the mean fluorescence intensity (MFI) within delineated islet areas was determined using the Mean Intensity function. MFI was measured in n = 6 biological replicates per group (day 15 post-treatment), across three non-overlapping islet areas per section (n = 18). Image acquisition and quantification were not performed under blinded conditions.

### 4.8. Statistical Analysis

Statistical analyses were performed using GraphPad Prism 8 (GraphPad Software, San Diego, CA, USA). Data normality was assessed using the Shapiro–Wilk test (α = 0.05), which is appropriate for the per-group sample size used here (n = 6). Outliers, if identified, were removed using Grubbs’ test (α = 0.05). Two analytical strategies were applied according to the structure of each dataset. First, longitudinal blood glucose, glycemic delta, and niAUC values, measured in the same animals on days 3, 6, 9, 12, and 15 post-treatment, were analyzed using two-way repeated-measures ANOVA with treatment and time as within-subject factors, followed by Tukey’s multiple comparisons test. Second, terminal endpoint variables (pVHL, HIF-1α, GLUT-1, GLUT-2, and insulin staining, quantified only on day 15) were analyzed using one-way ANOVA with treatment as the factor, followed by Tukey’s multiple comparisons test, since these readouts represent independent measurements rather than repeated measures. A *p*-value < 0.05 was considered statistically significant.

## 5. Conclusions

In this short-term, proof-of-concept study, a single intrapancreatic injection of an adenoviral vector encoding the murine *Vhlh* gene (AdVHL) into two genetically distinct mouse strains (C57BL/6 and BALB/c) with HCD-induced T2DM was associated with (i) a transient decrease in blood glucose values over a 15-day follow-up window, with the largest effect observed around days 9–12 post-injection; (ii) increased expression of pancreatic pVHL and a parallel reduction in HIF-1α and GLUT-1 expression; and (iii) an increase in GLUT-2 and insulin expression in pancreatic tissues. These findings support a functional role of the pVHL–HIF-1α axis in pancreatic tissues in a diet-induced (non-genetic) model of T2DM and provide proof-of-concept evidence that acquired, rather than inherited, reductions in pancreatic pVHL are reversible in the short term. This study is limited by (i) the short (15-day) follow-up window, (ii) a two-point glucose excursion measurement rather than a full glucose tolerance test, and (iii) the use of an adenoviral delivery route that is not clinically translatable. Accordingly, the present data suggest that the VHL–HIF-1α axis plays a functional role in the regulation of pancreatic glucose metabolism in both healthy and T2DM mice.

## Figures and Tables

**Table 1:** Experimental design for studying the effect of transient pVHL overexpression in pancreatic tissues of mice with T2DM.

Strain	Status	Treatment of the Sub-Groups in the Day 0	Glycemic Assessment (Δglucose & niAUC)	Day 15 (Histology Assay)
**C57BL/6 Group (n = 48)**	**T2DM** (n = 24)/4 Subgroups (n = 6)	**1)** Non-treated (n = 6) **2)** AdVHL 1 × 10^12^ vp/40 µL (n = 6) **3)** AdGFP 1 × 10^12^ vp/40 µL (n = 6) **4)** PBS (vehicle) 40 µL(n6)	*Day 0, Day 3 · Day 6 · Day 9 · Day 12 · and Day 15* *(to all the mice)*	*VHL, HIF1α, GLUT-1, GLUT2, and Insulin detection* (n = 6/Sub-group)
	**Healthy** (n = 24)/4 Subgroups (n = 6)			
**BALB/c Group (n = 48)**	**T2DM** (n = 24)/4 Subgroups (n = 6)	Administreated by Intrapancreatic via (to each Sub-group of mice)		
	**Healthy** (n = 24)/4 Subsgroups (n = 6)			

**vp = viral particles; Histological analysis (IHC/IF) performed at Day 15 only; Δglucose = Glycemic delta; niAUC= net incremental area under curve.**

## Data Availability

The original contributions presented in this study are included in the article. Further inquiries can be directed to the corresponding author.

## Abbreviations

The following abbreviations are used in this manuscript:

T2DM	Type 2 diabetes mellitus
pVHL	Von Hippel–Lindau protein
HIF-1α	Hypoxia-inducible factor 1-alpha
VEGF	Vascular endothelial growth factor
HCD	High-calorie diet
SD	Standard diet
MFI	Mean fluorescence intensity
AdVHL	Adenoviral vector encoding the murine *Vhlh* gene
AdGFP	Adenoviral vector encoding the *GFP* gene
PBS	Phosphate-buffered saline
IHC	Immunohistochemistry
IF	Immunofluorescence
UPR	Unfolded protein response
ER	Endoplasmic reticulum
niAUC	Net incremental area under the curve
